# Purity Assessment of Tripropyl Phosphate through Mass Balance and ^1^H and ^31^P Quantitative Nuclear Magnetic Resonance

**DOI:** 10.3390/molecules29091975

**Published:** 2024-04-25

**Authors:** Yuebing Wan, Kangcong Li, Xiuqin Li, Xiaomin Li, Hongtao Chu, Qinghe Zhang

**Affiliations:** 1 College of Chemistry and Chemical Engineering, Qiqihar University, Qiqihar 161006, China; 15088536404@163.com (Y.W.); lange1979@163.com (H.C.); 2 Division of Chemical Metrology and Analytical Science, National Institute of Metrology, Beijing 100029, China; 13097513253@163.com (K.L.); lixq@nim.ac.cn (X.L.); lixm@nim.ac.cn (X.L.); 3 Key Laboratory of Chemical Metrology and Applications on Nutrition and Health for State Market Regulation, Beijing 100029, China

**Keywords:** tripropyl phosphate, purity assessment, mass balance method, quantitative nuclear magnetic resonance

## Abstract

Tripropyl phosphate (TnPP) is a commonly used organic phosphate flame retardant in the textiles, plastics, and coating industries. Residues are commonly detected in samples from the environment and food. The availability of certified reference materials (CRMs) is essential to ensure the accuracy and traceability of detection results. In this study, a comprehensive characterization of a CRM for TnPP was carried out, and its purity was evaluated using two distinct methodologies: mass balance (MB) and quantitative nuclear magnetic resonance spectroscopy (qNMR). In the MB method, the levels of structurally related organic impurities are 1.37 mg/g. The water content was determined to be 3.16 mg/g, while inorganic impurities were found to be 0.87 mg/g, and no residual organic solvents were detected. Benzoic acid and monocrotophos were chosen as internal standards for ^1^H-qNMR and ^31^P-qNMR, respectively. The purity of the TnPP CRM was assessed as 994.6 mg/g, 994.1 mg/g, and 993.5 mg/g using MB, ^1^H-qNMR, and ^31^P-qNMR techniques, respectively. The verified purity of the TnPP CRM was ultimately determined to be 994.1 mg/g, with an expanded uncertainty of 3.4 mg/g (k = 2), ensuring traceability to the International System of Units (SI). This CRM can be effectively utilized for preparing calibration solutions suitable for the routine monitoring of TnPP residues in plastics and food samples.

## 1. Introduction

Organophosphorus flame retardants (OPFRs) represent a novel category of flame retardants, mainly including phosphinate, phosphonate, and phosphate ester compounds [1]. In comparison to traditional brominated flame retardants, OPFRs demonstrate reduced toxicity, enhanced corrosion resistance, and superior flame retardant and plasticizing properties. Considering the worldwide regulations limiting the use of brominated flame retardants, OPFRs have become prevalent in plastics, textiles, coatings, and various other materials [2], gradually displacing brominated flame retardants. As an additive flame retardant, it is usually incorporated into polymers through mechanical blending. Polymers exhibit high volatility and have the potential to be emitted into various environmental compartments, such as air [3,4], soil [5,6], and water [7,8,9,10], as a result of abrasion and leaching processes occurring during the production and utilization of raw materials. Eventually, they infiltrate the organism via biomagnification [11], thereby emerging as a noteworthy form of pollutant within the environmental domain [12,13,14].

Due to the frequent detection of organophosphate esters in the environment, there is a growing concern about the exposure routes and potential hazards in humans [15,16]. The research [17,18] shows that OPFRs are often ingested through dietary, breathing, and cutaneous exposures, with dietary intake being the primary route. In Western Europe, North America, and Asia, OPFRs have been detected in various foods, including cereals, vegetables, fish, meat, eggs, milk, desserts, and aquatic organisms. The detected levels vary significantly, ranging from below the lowest pg/g to above μg/g [19]. Countries have successively established standards for detecting tripropyl phosphate (TnPP) in the environment and food or conducted risk monitoring studies. Monitoring the residues of TnPP in the environment, food, and industrial products has attracted attention [20].

Metrological traceability is the foundation for ensuring the accuracy and comparability of measurements. Purity-certified reference materials (CRMs), typically positioned at the apex of the metrological traceability hierarchy, are essential for calibration solutions and measurements, serving as a crucial cornerstone in chemical measurements. Currently, there is a lack of a purity CRM of TnPP traceable to the International System of Units (SI). Therefore, there is an urgent need to develop a purity CRM for TnPP.

At present, the primary methods for determining the purity of organic compounds are mass balance (MB), quantitative nuclear magnetic resonance (qNMR), and differential scanning calorimetry (DSC). MB is an indirect method of measuring purity. It involves measuring the content of various types of impurities in a sample separately using different detection techniques and then deducting them from 100%. For instance, in high-purity organic samples, the content of the main component is determined by measuring structure-related organic impurities, water, residual organic solvents, and inorganic impurities separately and then subtracting these impurities from the total. The accuracy of MB depends on the baseline separation of impurities from the main component, as well as on the detection methods and instrumental sensitivity of various impurities. Gas chromatography (GC) and liquid chromatography (LC) are currently the primary methods for separating and analyzing the main component and impurities. To avoid potential overlap between the main component and impurities, as well as among impurities themselves, it is necessary to utilize at least two chromatographic columns and mobile phases with different properties for effective separation and comparison. This method [21] helps achieve baseline separation between various components and enhances the accuracy of the quantification. Steven Westwood et al. [22] systematically described the mass balance method to validate the measurement method for analyzing four types of impurities. Gong et al. [23] accurately determined the purity of folic acid using the mass balance method, while the water content was measured by enhancing the Karl Fischer method. Tu et al. [24] comprehensively summarized the types of domestic and international certified reference materials for neonicotinoid pesticides, along with sample preparation and determination techniques. Lee et al. [25] accurately evaluated the purity of the free base and salt forms of tetracycline hydrochloride using the mass balance method, with results traceable to SI units.

Quantitative nuclear magnetic resonance is a direct measurement method used to quantify purity. It relies on traceable CRMs as internal standards. This method is based on the principle that signal intensity is directly proportional to the number of nuclei generating a specific resonance [26,27]. With the advancement of instrumental technology and the enhancement of measurement accuracy and precision, qNMR has become increasingly common as a method for assessing organic purity. Currently, ^1^H spectra are the most commonly used qNMR method and have been incorporated into some pharmacopoeial guidelines and official standards. The Bureau International des Poids et Measures (BIPM) [28] has developed a set of eight internal standards for ^1^H spectra to assess the purity of organic compounds. Other one-dimensional spectra, such as P and F, where the analyte signals are usually less prone to impurity interference, have also been commonly used in food and pharmacology in recent years [29].

^31^P has a high gyromagnetic ratio (17.235 MHz·T^−1^), a wide range of chemical shifts [30], and a natural abundance of ^31^P nuclei of 100%, which provides excellent NMR sensitivity and high chemical shift dispersion. ^31^P-qNMR typically generates a single sharp peak corresponding to a single phosphorus atom, making it an excellent technique for studying phosphorus-containing compounds and for quantitative analysis. Compared to ^1^H-NMR, ^31^P-NMR avoids interference from complex matrices in most cases [31]. It produces sharp signals, the line shape is less affected by magnetic field inhomogeneities and eddy currents, and ^31^P-qNMR does not require expensive deuterated solvents, which simplifies sample preparation [32]. Studies [33] have shown that the accuracy and precision of ^31^P-qNMR methods are comparable to those of chromatographic methods, and they have been applied in drug and food analysis [34]. MANIARA et al. [35] systematically evaluated the parameters of precision, accuracy, specificity, linearity, limit of detection, and limit of quantification of ^1^H and ^31^P-qNMR. DEEN et al. [36] applied triphenyl phosphate and sodium phosphate as internal standards to determine the purity of phosphorus-containing compounds and compared them with ^1^H-NMR. Kato et al. [37] investigated the measurement of the absolute purity of synthetic phosphatidylcholine using ^31^P-qNMR, and the results were consistent with the determination using the internal standard ^1^H-qNMR.

In this investigation, commercially available high-purity TnPP was used as a candidate reference material. The compound was characterized using high-performance liquid chromatography coupled with high-resolution mass spectrometry (HPLC-HRMS) and NMR techniques. The purity was assessed using MB and qNMR techniques. This study utilized GC with a flame ionization detector (FID) and a flame photometric detector (FPD) for the separation and detection of compounds. Various analytical techniques were employed to quantify water content, inorganic impurities, and volatile solvents. In the assessment of purity using NMR, a novel approach was employed by combining ^1^H-NMR and ^31^P-NMR for the first time to determine the level of purity. Benzoic acid CRM was used as the internal standard for ^1^H-NMR, while monocrotophos CRM was utilized as the internal standard for ^31^P-NMR. The uncertainty associated with measuring purity was thoroughly assessed. This method enabled tracing purity back to SI units.

## 2. Results and Discussion

### 2.1. Qualitative Characterization

**Figure 1:**
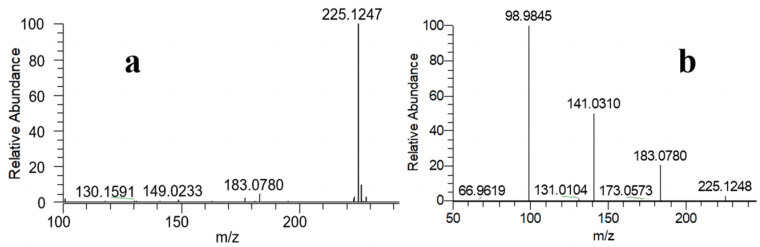
Qualitatively analyzed TnPP using HRMS: (**a**) the primary mass spectrum of TnPP; (**b**) the secondary mass spectrum of TnPP.

The objective of the qualitative characterization is to verify the distinctive structure of TnPP, which aligns with the molecular formula C_9_H_21_O_4_P, demonstrating a theoretically accurate mass-to-charge ratio of 225.1250. Through full-scan detection in the positive ion mode of the electrospray ionization source, TnPP typically generates quasi-molecular ions of [M + H]^+^. The precise mass-to-charge ratio observed in the primary mass spectrum of TnPP in Figure 1a is 225.1247. The deviation in precision from the theoretically accurate mass-to-charge ratio is −1.3326 ppm. The quasi-molecular ions were observed during high-energy collision-induced dissociation. Four significant characteristic ions (*m*/*z* = 225.1248, 183.0780, 141.0310, and 98.9845) were identified in the secondary mass spectrum of TnPP, as illustrated in Figure 1b. Due to the molecular weight of triethyl phosphate (TEP) being 182.0708, in order to exclude the possibility that the mass-to-charge ratio of 183.0780 was TEP, the retention time of impurities in TnPP (10.750 min) was compared with TEP (8.784 min) by GC-FID, and TEP was not found to be present. The ions detected in the mass spectrum corresponded to [M + H]^+^, [M − R + 2H]^+^, [M − 2R + 3H]^+^, and [M − 3R + 4H]^+^, where R represents C_3_H_7_. The ratio of relative abundance between [M − 3R + 4H]^+^ (*m*/*z* = 98.9845) and [M − 2R + 3H]^+^ (*m*/*z* = 141.0310) is 2:1.

**Figure 2:**
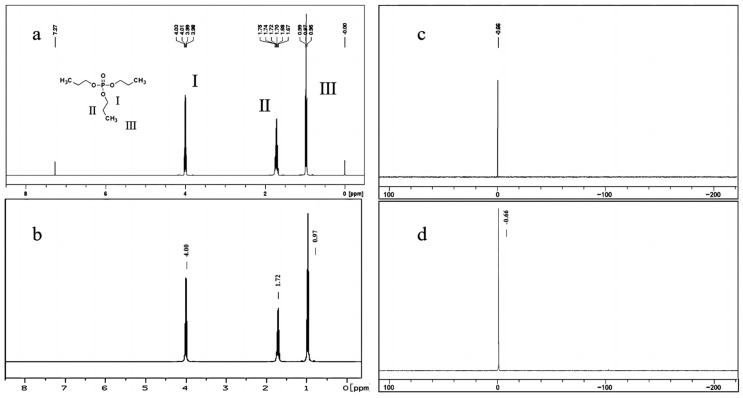
Qualitatively analyzed TnPP using the ^1^H-NMR spectrum and ^31^P-NMR spectrum: (**a**) ^1^H-NMR spectrum of TnPP in deuterated chloroform solvent; (**b**) ^1^H-NMR spectrum of TnPP in deuterated chloroform solvent in the SDBS; (**c**) ^31^P-NMR spectrum of TnPP in deuterated chloroform solvent; (**d**) ^31^P-NMR spectrum of TnPP predicted by MestReNova.

Figure 2a illustrates the ^1^H-NMR spectrum of TnPP in deuterated chloroform, exhibiting a single peak at a chemical shift of 7.27 ppm, which is attributed to the solvent. Additionally, at 4.00 ppm, there are two hydrogens at the I position on TnPP, exhibiting a quartet signal with a coupling constant of 6.7 Hz. These hydrogens are coupled to both the hydrogens situated at the II position and to phosphorus. The peaks observed at 1.71 ppm (m, 2H) and 0.97 ppm (t, *J* = 7.4 Hz, 3H) correspond to the hydrogen atoms located on the CH_2_ and CH_3_ groups at the II and III positions, respectively. This is consistent with the ^1^H-NMR spectrum of TnPP as provided by the Spectral Database System (SDBS) [38] in Figure 2b. The ^31^P-NMR spectrum of TnPP is depicted in Figure 2c. The singular peak observed at −0.66 ppm corresponds to the phosphorus peak from TnPP, which is consistent with the expected spectrum shown in Figure 2d of MestReNova (V14.2). The qualitative results presented above clearly indicate that the candidate material is TnPP.

### 2.2. Quantitative Analysis by MB

#### 2.2.1. Determination of TnPP and Its Structurally Related Impurities by GC-FID

The identification of structurally related impurities through GC techniques is an essential component of MB. The presence of impurities is primarily associated with the industrial synthesis process. The primary obstacle in identifying structurally related impurities is achieving baseline separation between the main component and individual impurities. The optimal chromatographic conditions were determined by considering factors such as the number of impurity peaks, the symmetry of the main peak, and the separation of the main component from the impurity peaks. This was achieved through the optimization of sample injection concentration, chromatographic column selection, inlet temperature, and the heating procedure.

To improve the efficiency of gasification for both the main component and impurities, this study compared the influence of three inlet temperatures (260 °C, 280 °C, and 300 °C) on the levels of TnPP and impurities. The result indicated the presence of six impurities across the three inlet temperatures, with impurity levels varying from 0.03 mg/g to 0.70 mg/g. The relative content of impurities exhibited no significant variation with changes in inlet temperatures (Figure S1), except for impurity 3, which increased from 0.57 mg/g to 0.70 mg/g (Table S1). Considering the column wastage and other factors, an inlet temperature of 280 °C was chosen for the experiment.

This study examined the impact of three different heating rates (5 °C/min, 10 °C/min, and 20 °C/min) on the isolation and content of TnPP, including its impurities. The results indicated the presence of TnPP and six impurities when the temperature was increased at a rate of 20 °C/min. Nevertheless, only five impurities were detectable when the temperature was increased at rates of 5 °C/min and 10 °C/min. The observed phenomenon can primarily be attributed to the broadening of the peaks and the increase in tailing at lower heating rates, which lead to a decrease in separation efficiency. In conclusion, a heating rate of 20 °C/min was selected for the procedure.

The DB-1MS UI, DB-5MS UI, and DB-17MS columns were compared in the separation. No obvious differences have been found between the DB-5MS UI and DB-1MS UI columns. Despite this, the DB-17MS column only succeeded in separating five impurities, and the separation was considered unsatisfactory. The n-hexane solutions containing TnPP at concentrations of 100, 300, 500, 700, 900, and 1000 μg/mL were prepared. The impurity peaks and purity measurements of TnPP used the DB-1MS column with dimensions of 30 m × 0.32 mm × 1.0 μm and 30 m × 0.25 mm × 0.25 μm. The response and linearity of both the main component and impurities were found to be satisfactory across all concentration ranges when using the 0.32 mm × 1.0 μm column (Figure S2). When using the 0.25 mm × 0.25 μm column, the main peak displayed a flat head at 1000 μg/mL, indicating a deviation from linearity. In conclusion, using a column with a larger inner diameter and a thicker film can increase the column capacity and decrease the risk of column overloading. The DB-1MS capillary column (30 m × 0.32 mm × 1.0 μm) was chosen for the analysis.

Under identical chromatographic conditions, a comparison was made between the signals of impurities in the FID and the FPD, as illustrated in Figure S3a,b. It is evident that the impurity eluting at 7.926 min in the FID chromatogram may align with the impurity eluting at 7.950 min in the FPD chromatogram, suggesting the presence of the element of phosphorus in the impurity. The area-normalized purity of the FID was determined to be 998.63 mg/g, slightly higher than the FPD purity of 998.10 mg/g. The levels of structurally related impurities are 1.37 mg/g and 1.90 mg/g, respectively.

#### 2.2.2. Water Determination

Water is ubiquitous in the environment and present in most samples. The precise measurement of water content and verification of uncertainty are crucial aspects of MB. Karl Fischer titration is considered a significant quasi-baseline technique for determining the water content in organic substances, providing results that are traceable to SI units. TnPP exhibits good solubility in methanol, making the direct injection method feasible. A blank was determined through virtual addition to account for external water introduced during sample measurement. Subsequently, the water content value determined for TnPP was adjusted by subtracting the blank value. The results indicated that the water content was 3.16 mg/g, with a relative standard deviation (RSD) of 6.4% (*n* = 6, Table 1).

#### 2.2.3. Inorganic Impurity Determination

A semi-quantitative analysis employing ICP-MS in He mode was performed to detect seventy inorganic elements. The concentration of total inorganic impurities was measured at 0.87 mg/g. The elements exhibited the highest concentrations include Mg (0.605 mg/g), Ti (0.097 mg/g), and Na (0.093 mg/g) (Table S2).

#### 2.2.4. Residual Organic Solvent Determination

Residual solvents found in samples of candidate materials typically originate from organic volatile compounds that have been used but not removed during the process of sample preparation. The analysis of residual organic solvents was performed utilizing the HS-GC/FID method. In this procedure, TnPP was dissolved in DMSO, along with twelve frequently used solvents also dissolved in DMSO. The identification of residual solvent types relied on GC retention time, while their quantities were determined using the external standard method. The findings indicated the absence of organic solvent residues in TnPP.

#### 2.2.5. Mass Fraction by MB

In the assessment of TnPP purity related to MB, the equation is formulated as shown in Equation (1) as follows:

(1) $$\begin{array}{c} P_{MB}=P_{GC}\times \left(1000-X_{W}-X_{V}-X_{NV}\right) \end{array}$$

where *P_MB_* represents MB purity, *P_GC_* is TnPP purity determined by GC area normalization, which is 998.63 mg/g, *X_W_* is water content, measured at 3.16 mg/g, *X_NV_* is inorganic impurity content, recorded at 0.87 mg/g, and *X_V_* is volatile solvent content, noted as 0.0 mg/g.

The final purity of TnPP was determined to be 994.60 mg/g (Table S3).

### 2.3. Quantitative Analysis by qNMR

The utilization of qNMR as a precise quantitative technique to evaluate the purity of TnPP resolves the limitation of the MB method, which might not identify all impurities. The accuracy of the qNMR technique relies on employing an SI-traceable internal standard CRM to ensure precise quantification. Moreover, it is essential to ensure that the internal standard selected does not display spectral peaks that overlap with those of TnPP to prevent any interference in the measurements. In this study, benzoic acid and monocrotophos were chosen as internal standards due to their compatibility with the established criteria. Benzoic acid exhibits four distinct stable hydrogen chemical shifts, while monocrotophos demonstrates a stable phosphorus chemical shift. Importantly, none of these shifts overlap with the spectral peaks of TnPP. For the purity assessment, TnPP and either benzoic acid or monocrotophos were accurately weighed and dissolved in DMSO-D_6_. This process enables the precise determination of the purity of TnPP through qNMR analysis.

The equation for qNMR purity assessment can be represented as follows:

(2) $$P_{TnPP}=\frac{I_{TnPP}}{I_{Std}}\frac{n_{Std}}{n_{TnPP}}\frac{M_{TnPP}}{M_{Std}}\frac{m_{Std}}{m_{TnPP}}P_{Std}$$

where *P_TnPP_* and *P_std_* are the purity of TnPP and internal standard (mg/g), respectively, *m_TnPP_* and *m_std_* are the weight of TnPP and internal standard (g), respectively, *M_TnPP_* and *M_std_* are the molecular weight of TnPP and internal standard (g/mol), respectively, *I_TnPP_* and *I_std_* are the integrated signal intensities of quantification peaks of TnPP and internal standard, respectively, and *n_TnPP_* and *n_std_* are the number of hydrogens in the quantification peaks of TnPP and internal standard, respectively.

The equation includes the ratio of the integrated peak areas for TnPP and the internal standard, the weights of TnPP and the internal standard added to the NMR tube, their respective molecular weights, and the number of resonating hydrogens contributing to the observed peaks. The rationale of multiplying by 1000 is to facilitate the conversion of purity values from fractional form to milligrams per gram (mg/g), enabling a direct representation of TnPP purity in the commonly used milligram per gram format. This qNMR method provides a precise and traceable approach to assessing the purity of substances. It significantly benefits from its accuracy and the incorporation of internal standards, which ensure traceability to the SI units.

#### 2.3.1. Quantitative Analysis by ^1^H-qNMR

**Figure 3:**
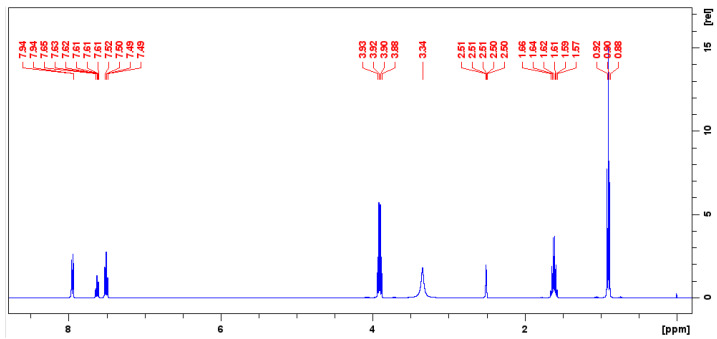
Quantitative hydrogen spectrum of a mixture of benzoic acid and TnPP in deuterated DMSO.

The purity of TnPP was accurately assessed using ^1^H-qNMR, with benzoic acid used as the CRM for traceability. This approach utilizes the relative integration ratios of specific hydrogen signals from both benzoic acid and TnPP. The selection of benzoic acid as an internal standard is crucial due to its well-defined and stable peak characteristics, which are essential for attaining high accuracy in quantitative NMR studies. From the ^1^H-NMR spectrum depicted in Figure 3, the hydrogen signal of benzoic acid at 7.95 ppm and the TnPP hydrogen signal at 4.00 ppm exhibited significant distinctiveness and were well separated from the remaining spectral peaks. This distinct separation displayed excellent symmetry, which is essential for accurate integration. The selection of these two hydrogen peaks for quantification highlights the significance of distinct, separate peaks in quantitative NMR analysis. This can reduce interference and ensure accurate results. Applying Equation (2) for the calculation of purity, the purity of TnPP was determined to be 994.1 mg/g. The figure was derived from seven repeated measurements, indicating the method’s high reliability and precision, as evidenced by the notably low RSD of 0.12%. The low RSD value emphasizes the reproducibility and accuracy of the qNMR method when utilized in conjunction with a suitable internal standard, such as benzoic acid and carefully chosen hydrogen signals for quantification.

#### 2.3.2. Quantitative Analysis by ^31^P-qNMR

Uchiyama et al. [39] found that the qNMR parameters had a significant effect on the determination results, and they improved the accuracy of the measurement process by optimizing the qNMR parameters. They also compared the determination of the purity content of cyclophosphamide hydrate and sofosbuvir employing ^1^H-qNMR and ^31^P-qNMR, and the analysis results indicated that two different qNMR determinations were matched. Therefore, the accuracy of ^31^P-qNMR was improved in this study by optimizing the qNMR parameters. The evaluation of the purity of TnPP utilizing ^31^P-qNMR demonstrates the effectiveness of the method when monocrotophos is used as a CRM for traceability, with a particular focus on phosphorus signals. The selection of monocrotophos as an internal standard lies in its unique and consistent phosphorus signal, which improves the accuracy of the quantification process. Uchiyama et al. [40] utilized ^31^P-qNMR to determine the purity of brigatinib, and the result was consistent with that measured by ^1^H-qNMR, validating the possibility of ^31^P-qNMR for the measurements of phosphorus-containing compounds, and this study is identical to its results.

**Figure 4:**
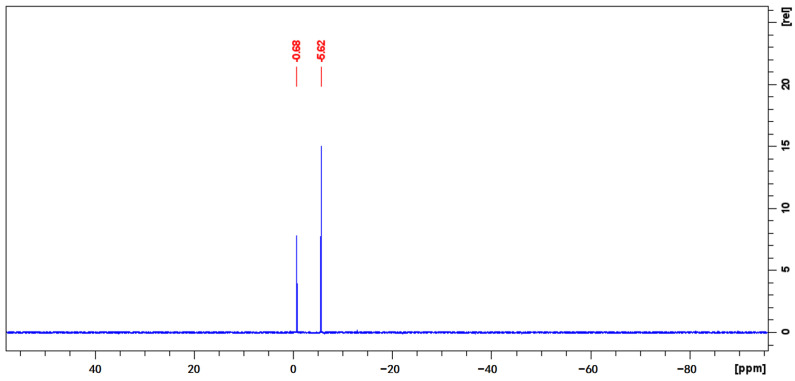
Quantitative phosphorus spectrum of a mixture of monocrotophos and TnPP in deuterated DMSO.

In the ^31^P-qNMR spectrum illustrated in Figure 4, the phosphorus signal of monocrotophos is detected at −5.62 ppm, while TnPP is observed at −0.68 ppm. These signals represent the only peak within their specific regions, ensuring clear identification and separation from any potential signal overlaps. The significant peak symmetry observed in both signals is crucial for achieving accurate integration, which is a cornerstone for reliable qNMR analysis. The selection of these specific phosphorus peaks for the quantification process aligns with the principle of choosing distinct, non-overlapping signals to ensure the highest levels of accuracy in qNMR purity evaluations. The application of Equation (2) yielded a calculated purity of 993.5 mg/g for TnPP (Table S4). This result was consistently replicated in seven measurements, resulting in a remarkably low RSD of 0.11%. It is crucial that the application of ^31^P-qNMR avoids interferences, yields a single and sharp phosphorus peak, and is consistent with the result of ^1^H-qNMR.

The utilization of qNMR for purity assessment, especially when supported by a CRM, like benzoic acid and monocrotophos, offers a precise, consistent, and verifiable approach for evaluating the purity of compounds, such as TnPP. The effectiveness of this method is based on its direct correlation with the number of nuclei involved in the resonance, making it a valuable tool in evaluating purity and overseeing quality control procedures.

### 2.4. Value Assignment

The minor difference in purity results derived from two distinct methods is a common phenomenon, where the MB method yields slightly higher values compared to qNMR. This discrepancy arises from the intrinsic constraints of each method in accurately quantifying impurities. While the MB method may overestimate purity due to incomplete quantification of impurities, qNMR provides increased precision and accuracy. However, qNMR may overlook specific impurities. In accordance with ISO 33407 [41], the purity of the TnPP CRM, determined using the MB and qNMR methods, was cross-checked via a *t*-test and an F-test. The value of the F-test was less than F_0.05(22,2)_, and the *t*-test was less than *t*_0.05(16)_, which indicated that the two methods have equal accuracy (Table 2). Consequently, the final purity result of the TnPP CRM should be calculated from the average of the above two independent methods, which was determined to be 994.1 mg/g. This value signifies a comprehensive evaluation that considers the combined advantages and limitations of both the MB and qNMR methodologies. This approach guarantees that the ultimate purity outcome mirrors a more thorough and comprehensive understanding of the substance’s purity, reducing the influence of biases specific to the method. It also provides a stronger foundation for quality assurance and certification of reference materials.

### 2.5. Uncertainty Evaluation

The assessment of uncertainty adheres to the guidelines outlined in JJF 1855-2020 [42] and ISO Guide 35:2017 [43]. These documents offer a systematic approach to assessing uncertainty by considering input contributions from both MB and qNMR methods, as well as their main influencing factors (Table 3 and Table 4). The combined standard uncertainty (*u_MB_*) for MB is calculated using a quadratic combination of uncertainties associated with all detectable impurities. This process involves considering four primary factors: (1) uncertainty linked to the organic purity determined by GC, (2) uncertainty associated with water content, (3) uncertainty associated with volatile solvent content, and (4) uncertainty related to inorganic impurity content, as represented in Equation (3) as follows:

(3) $$u_{MB}=\sqrt{{u^{2}}_{GC}+{u^{2}}_{W}+{u^{2}}_{V}+{u^{2}}_{NV}}=2.1\;mg/g$$

where *u_GC_* is the standard uncertainty associated with the organic purity determined by GC, which is 0.21 mg/g, *u_W_* is the standard uncertainty associated with water content, recorded at 2.1 mg/g, *u_V_* is the standard uncertainty associated with volatile solvent content, noted as 0.0 mg/g, and *u_NV_* is the standard uncertainty associated with inorganic impurity content, measured at 0.2 mg/g.

In this instance, the combined standard uncertainty, denoted as *u_MB_*, is calculated to be 2.1 mg/g. This value represents the overall uncertainty associated with the MB method, taking into account various contributing factors.

In Equation (4), we obtain the following:

(4) $$u\left(P_{NMR}\right)=P_{NMR}\sqrt{{\left(\frac{u\left(I_{x}/I_{std}\right)}{I_{x}/I_{std}}\right)}^{2}+{\left(\frac{u\left(M_{x}\right)}{M_{x}}\right)}^{2}+{\left(\frac{u\left(M_{std}\right)}{M_{std}}\right)}^{2}+{\left(\frac{u\left(m_{x}\right)}{m_{x}}\right)}^{2}+{\left(\frac{u\left(m_{std}\right)}{m_{std}}\right)}^{2}+{\left(\frac{u\left(P_{std}\right)}{P_{std}}\right)}^{2}}$$

where *I_x_*/*I_std_* is the uncertainty of the integral area measurements ratio (mg/g), *M_x_* and *M_std_* are uncertainties of the relative molecular weight of the sample and internal standard (g/mol), *m_x_* and *m_std_* are uncertainties of the weighing sample and internal standard on balance (mg), *P_std_* is the uncertainty of purity for the internal standard (mg/g), and *P_TnPP-NMR_* is uncertainties of ^1^H-qNMR/^31^P-qNMR measurements (mg/g).

The equivalence in precision of these two methods necessitates the calculation of the uncertainty of the standard value using Equation (5) as follows:

(5) $$u_{char}=\frac{\sqrt{\left({u^{2}}_{MB}+{u^{2}}_{1H-qNMR}+{u^{2}}_{31p-qNMR}\right)}}{3}=\frac{\sqrt{\left(2.1^{2}+1.6^{2}+4.2^{2}\right)}}{3}=1.7\;mg/g$$

Upon completion of the analysis, the expanded uncertainty (*U_char_*) was calculated by multiplying the combined standard uncertainty (*u_char_*) by an expanded factor of 2 at a 95% confidence level. The expanded uncertainty was consequently determined to be 3.4 mg/g (k = 2).

## 3. Materials and Methods

### 3.1. Chemicals and Materials

TnPP candidate was purchased from Sigma-Aldrich (St. Louis, MO, USA). HPLC grade n-hexane was purchased from Fisher Chemical (Waltham, MA, USA). Benzoic acid CRM (GBW06148, 99.991 ± 0.024%, k = 2), monocrotophos CRM (GBW06409, 99.9 ± 0.2%, k = 2), and water content CRM (GBW13517, 50.7 ± 0.6 mg/g, k = 2) were obtained from the National Institute of Metrology (Beijing, China). Deuterated chloroform for the NMR spectra was provided by J&K Scientific (Beijing, China). Seventy semi-quantitative calibration standard solutions for inorganic elements were obtained from Agilent Technology (Tokyo, Japan). The pure raw material of TnPP was prepared as a stock solution at a concentration of 1.0 mg/mL in n-hexane and stored at 4 °C away from light.

### 3.2. Instruments

The Agilent GC-7890A (Santa Clara, CA, USA) was equipped with FID and FPD for the analysis of major constituents and structurally relevant impurities. The chromatographic columns used were Agilent J&W DB-1 (30 m × 0.32 mm × 1.00 μm) and DB-5 (30 m × 0.25 mm × 0.25 μm). The Agilent GC-6890N (Santa Clara, CA, USA) was equipped with an FID and a G1888-headspace injector for quantifying residual organic solvents. The chromatographic column used was an Agilent J&W DB-624 (30 m × 0.32 mm × 1.80 μm). The Thermo Q Exactive Plus (Waltham, MA, USA) was equipped with a heated electrospray ionization source (HESI) for the mass spectrometric characterization of TnPP.

Bruker AVANCE III 400 MHz NMR (Billerica, MA, USA) was used for characterization and quantification. Data processing was conducted using Bruker NMR software TopSpin 3.1.

Water content was determined using a METTLER TOLEDO C30S Karl Fischer titrator (Greifensee, Switzerland). Inorganic impurities were analyzed and determined using an Agilent 8800 ICP-MS (Tokyo, Japan). The samples were weighed using a METTLER TOLEDO XP205 or UMX2 balance (Greifensee, Switzerland). A Vortex-Genie2 vortex oscillator G560E was used to prepare homogenized process solutions (Scientific Industries, Inc., Bohemia, NY, USA).

### 3.3. Qualitative Characterization

#### 3.3.1. LC-MS/MS Analysis

TnPP was analyzed using HRMS equipped with an HESI. The data were collected through flow injection at a constant rate of 10 μL/min. Simultaneously, the blank solvent was analyzed to prevent any influence from the solvent background. The specific parameters were as follows: positive ion scanning mode, selecting the full scanning of primary mass spectrometry, and parallel reaction detection mode (Full MS/PRM). The mass scanning range of the parent ion was *m*/*z* 100~500. In the primary mass spectrometry, the resolution was 70,000, while in the secondary mass spectrometry, it was 35,000. The collision energies were set at 10, 40, and 80 eV.

#### 3.3.2. ^1^H-NMR and ^31^P-NMR Qualitative Analysis

A total of 3.0 mg of TnPP was dissolved in 0.5 mL of deuterated chloroform. The resulting clear solution was transferred to a 5 mm NMR tube, which was then placed into the NMR instrument using an autosampler. The NMR probe head was maintained at 299 K, and the data were processed using TopSpin 3.1.

^1^H-NMR conditions: an excitation pulse angle of 90°, a relaxation delay of 43.96 s, and a zg pulse sequence for data acquisition. The data were acquired as 64 K time domain data points with 128 cumulative samples.

^31^P-NMR conditions: an excitation pulse angle of 90°, a relaxation delay of 53.69 s, and a zgpg pulse sequence for data acquisition. The data were acquired as 64 K time domain data points with 128 cumulative samples.

### 3.4. Quantitative Analysis by MB

#### 3.4.1. Structurally Related Impurities Determination

The quantification of structurally related impurities was conducted through GC-FID and GC-FPD. A blank solvent injection was utilized to reduce the influence of solvent background interference. The purity of TnPP was determined through the area normalization method. The structurally related impurities were analyzed using Agilent J&W DB-5 (30 m × 0.25 mm × 0.25 μm) and DB-1 MS (30 m × 0.32 mm × 1.0 μm) columns. Nitrogen (99.999%) was employed as the carrier gas at a flow rate of 1.0 mL/min. The temperature of the inlet was set at 280 °C, while the detector temperature was held constant at 300 °C. The injection volume was 1 μL, and the splitless injection mode was employed. The heating procedure involved an initial hold at 50 °C for 1 min, followed by a gradual temperature ramp to 300 °C at a rate of 20 °C/min, and ultimately a 2 min maintenance of the temperature at 300 °C.

#### 3.4.2. Water Determination

Residual water content was determined using a METTLER TOLEDO C30S Karl Fischer titrator (Columbus, OH, USA) via the direct injection method. The water content in the CRM was initially accurately weighed and determined to verify the accuracy of the measurement process. TnPP (approximately 50 mg) was then weighed and quickly added, and water content was determined. The measurement was repeated six times. To eliminate the influence of water in the air on the measurement results, the sampling process and conditions were simulated. A blank value for water in the air was calculated and subtracted from the measured value of the sample.

#### 3.4.3. Inorganic Impurity Determination

Semi-quantitative analyses of 70 common elements were carried out using ICP-MS (Agilent Technologies, Inc., Tokyo, Japan). The instrument parameters were set as follows: sampling depth 8 mm, pump speed 0.1 rps, RF power 1550 W, nebulizer temperature 2 °C, carrier gas flow rate 0.8 L/min, optional gas 20% argon–oxygen mixture (20% oxygen), and reaction mode He mode. The semi-quantitative factor curve of the instrument was calibrated using multi-element calibration standard solutions provided by Agilent. This calibrated curve was used to determine the value of samples with unknown concentrations of elements, and the measurements were repeated five times. To avoid interference from impurities in the solvent, the response of the blank solvent was determined, and the background value was subtracted from the elemental response of the sample.

#### 3.4.4. Residual Organic Solvent Determination

The residual organic solvents were analyzed using GC-FID (Santa Clara, CA, USA) equipped with a headspace injector. The experimental parameters were as follows: the inlet temperature was 130 °C, with splitless injection. Nitrogen (99.999%) was used as the carrier gas at a flow rate of 1.0 mL/min. The initial temperature of the heating block was 40 °C and was maintained for 5 min. Subsequently, the temperature was increased to 100 °C at a rate of 5 °C/min, held for 1 min, and then raised to 220 °C at 15 °C/min, and maintained for 1 min. The total duration of the run was 27 min. The equilibrium temperature of the headspace, the sample quantification loop temperature, and the transfer line temperature were 90 °C, 115 °C, and 120 °C, respectively. A total of 100 mg of TnPP was weighed into a 20 mL headspace vial. Subsequently, 10 mL of DMSO was added to dissolve the substance, and the mixture was shaken and mixed thoroughly. Finally, it was transferred into the headspace injection device for analysis. To eliminate interference from the solution, the response of each organic solvent in the blank solution was determined and subtracted from the sample.

### 3.5. Quantitative Analysis by qNMR

#### 3.5.1. Quantitative Analysis by ^1^H-qNMR

The purity of TnPP was analyzed using a 400 MHz NMR instrument (Billerica, MA, USA) with benzoic acid CRM as the internal standard. The accurately weighed sample and the internal standard, benzoic acid, were placed in the same brown glass vial. Deuterated DMSO was added and shaken to dissolve them completely. Subsequently, they were transferred to a 5 mm NMR tube. The NMR tube containing the samples to be measured and the internal standard was then placed into the NMR instrument using the autosampler. The specific parameters were as follows: excitation pulse angle of 90°, time domain data point of 64 K, excitation center (O_1_P) at 5.98 ppm, relaxation delay (D_1_) of 43.96 s, 128 cumulative samples, probe temperature at 299.0 K, pulse sequence zg, and quantification peaks at 4.00 ppm (TnPP) and 7.95 ppm (benzoic acid).

#### 3.5.2. Quantitative Analysis by ^31^P-qNMR

The purity of TnPP was analyzed using a 400 MHz NMR instrument with monocrotophos CRM as the internal standard. The accurately weighed sample and the internal standard monocrotophos were placed in the same brown glass vial. Deuterated DMSO was added and shaken to dissolve it completely, Subsequently, it was transferred to a 5 mm NMR tube. The NMR tube containing the samples to be measured and the internal standard was then inserted into the NMR instrument for detection using the autosampler. The specific parameters were as follows: excitation pulse angle of 90°, time domain data point of 64 K, excitation center (O_1_P) at −3.15 ppm, relaxation delay (D_1_) of 53.69 s, 128 cumulative samples, probe temperature at 299.0 K, pulse sequence zgpg, and quantification peaks at −0.62 ppm (TnPP) and −5.68 ppm (monocrotophos).

## 4. Conclusions

The thorough investigation of the TnPP CRM for SI-traceable purity assessment has been successfully concluded. The structural features of TnPP were verified using qualitative analysis techniques, such as ^1^H-NMR, ^31^P-NMR, and mass spectrometry. Subsequent purity determination using both MB and qNMR methods has resulted in a purity value of 994.1 mg/g, with an expanded uncertainty of 3.4 mg/g (k = 2). In the MB method, the levels of structurally related organic impurities are measured at 1.37 mg/g. The water content was determined to be 3.16 mg/g, whereas the inorganic impurity content was assessed at 0.87 mg/g. Notably, no residual organic solvents were detected. In the qNMR methodology, benzoic acid CRM was chosen as the internal standard for ^1^H-qNMR, while monocrotophos CRM was chosen for ^31^P-qNMR. The selection of internal standards ensures accurate quantification of TnPP purity, with careful consideration given to the non-overlapping hydrogen peaks between TnPP and the internal standards. The established purity value of TnPP can be accurately linked to the SI units through a continuous calibration chain, ensuring the highest precision and traceability. The new TnPP CRM signifies a notable advancement, providing laboratories with a chance to achieve more accurate and dependable results in their pursuits. The provision of this CRM promotes the monitoring of TnPP in the fields of environment and food, improves quality assurance practices, and ensures consistency across laboratories. It is crucial to store the TnPP CRM under suitable conditions to better ensure its characteristic values.

## Figures and Tables

**Table 1:** Water content determined using the Karl Fischer method.

Number of Measurements	Relative Content (mg/g)	Average Value (mg/g)	RSD (%)
1	2.84	3.16	6.40
2	2.99		
3	3.23		
4	3.26		
5	3.31		
6	3.35		

**Table 2:** Equal accuracy test of different methods.

Number of Measurements	MB (mg/g)	^1^H-qNMR (mg/g)	^31^P-qNMR (mg/g)
1	994.60	994.7	992.6
2	994.60	992.6	993.7
3	994.61	993.6	994.6
4	994.60	995.7	994.3
5	994.59	994.8	992.0
6	994.60	992.7	994.5
7	994.59	994.4	992.7
8	994.58	-	-
9	994.60	-	-
10	994.60	-	-
11	994.59	-	-
Mean	994.60	994.1	993.5
SD ^1^	0.008	1.151	1.048
*F*_calculate_	3.41		
*F*_0.05(22,2)_	3.44		
Conclusion	*F*_calculate_ < *F*_0.05(22,2)_, the means are equal		
*t*_calculate_	1.54		
*t*_0.05(16)_	2.12		
Conclusion	*t*_calculate_ < *t*_0.05(16)_, the SDs are equal		

^1^ Standard deviation.

**Table 3:** Uncertainties introduced by the MB method.

Components	Sources of Uncertainty	Mass Fraction (mg/g)	Standard Uncertainty (mg/g)
*u_GC_*	*P_GC_*	998.63	0.21
*u_W_*	*X_W_*	3.16	2.10
*u_V_*	*X_V_*	0.00	0.00
*u_NV_*	*X_NV_*	0.87	0.20
*u_MB_*	*P_MB_*	994.60	2.10

**Table 4:** Uncertainties introduced by qNMR.

Components	Sources of Uncertainty	^1^H-qNMR Mass Fraction (mg/g)	^1^H-qNMR Uncertainty (mg/g)	^31^P-qNMR Mass Fraction (mg/g)	^31^P-qNMR Uncertainty (mg/g)
*u(* *I_x_/I_std_)*	*I_x_/I_std_*	994.1	1.2	993.5	1.0
*u(* *M_x_)*	*M_x_*	224.2368	0.0043	224.2368	0.0043
*u(* *M_std_)*	*M_std_*	122.1230	0.0033	223.1648	0.0034
*u(* *m_x_)*	*m_x_*	3.003	0.002	3.003	0.002
*u(* *m_std_)*	*m_std_*	2.000	0.002	2.000	0.002
*u(* *P_std_)*	*P_std_*	999.9	0.2	999.0	4.0
*u(* *P_TnPP-NMR_)*	*P_TnPP-NMR_*	994.1	1.6	993.5	4.2

## Data Availability

Data are contained within the article and Supplementary Materials.

## Supplementary Materials

The following supporting information can be downloaded at: https://www.mdpi.com/article/10.3390/molecules29091975/s1, Figure S1: Content of impurities detected at different inlet temperatures; Figure S2: Purity of TnPP determined at different concentrations; Figure S3: Gas chromatogram of TnPP using different detectors: (a) gas chromatogram of TnPP by FID and (b) gas chromatogram of TnPP by FPD; Table S1: Comparison of the major component and impurity content at different inlet temperatures; Table S2: Inorganic impurity content in TnPP; Table S3: Purity results of TnPP by the MB method; Table S4: Purity results of TnPP by qNMR.
