# Supplementary material for: Purity Assessment of Tripropyl Phosphate through Mass Balance and 1H and 31P Quantitative Nuclear Magnetic Resonance
Source: Molecules. 2024 Apr 25;29(9):1975. doi: 10.3390/molecules29091975 (PMC11085218; doi:10.3390/molecules29091975)
Supplement: Supplementary file 1 [file molecules-29-01975-s001.zip › molecules-2954216-supplementary.pdf]

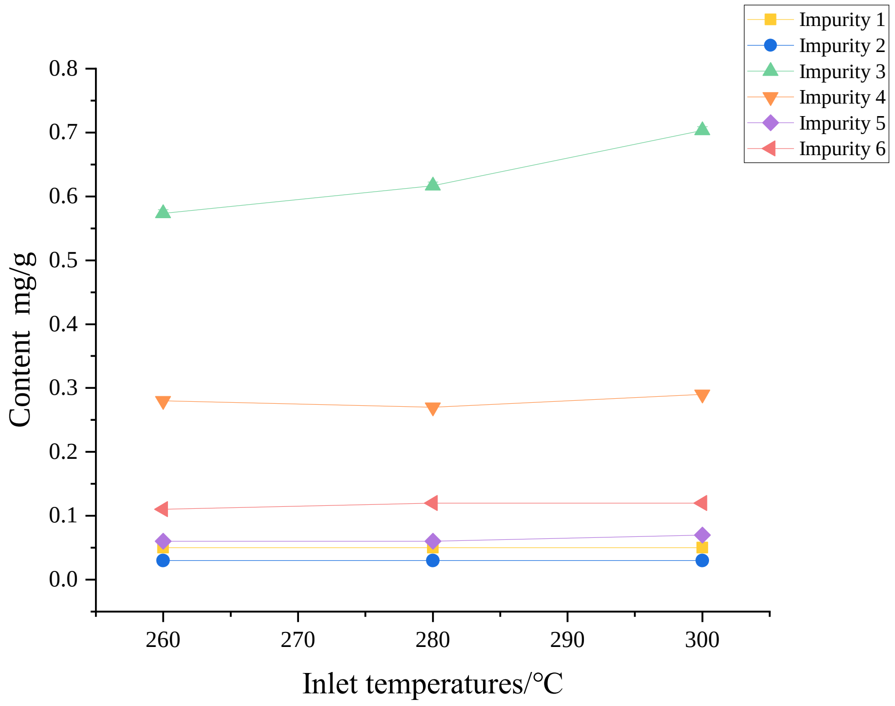

Figure S1. Content of impurities detected at different inlet temperatures

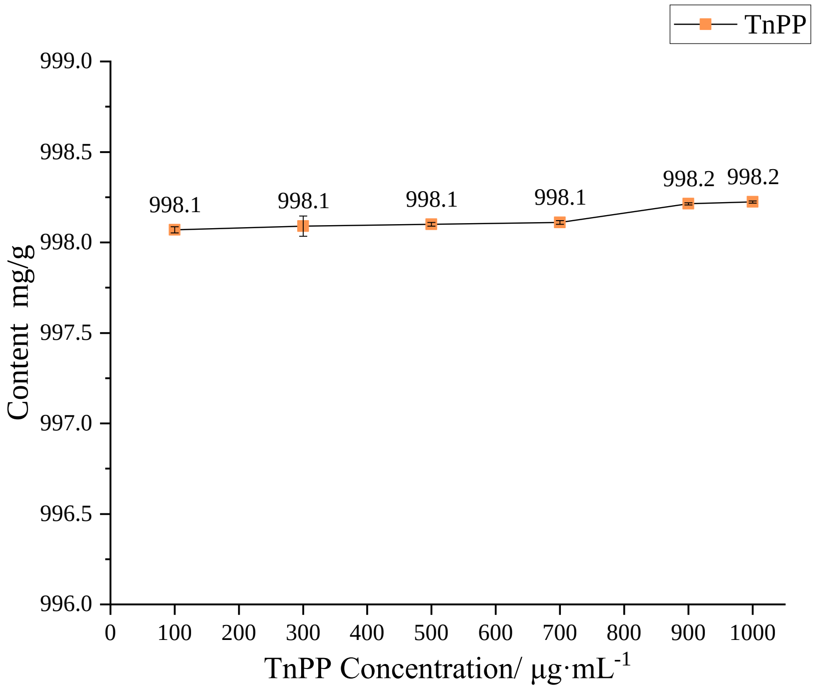

Figure S2. Purity of TnPP determined at different concentrations

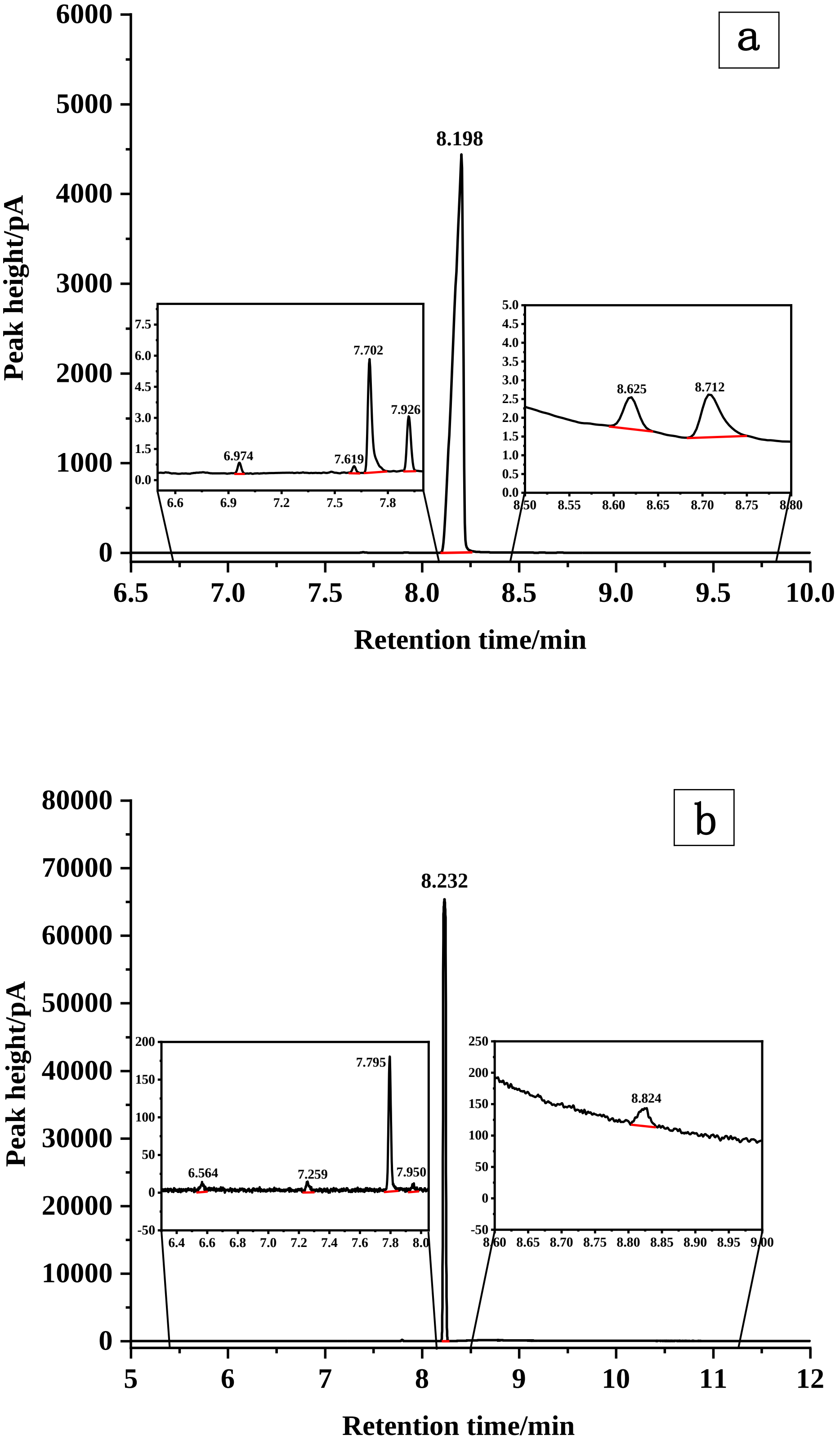

Figure S3. Gas chromatogram of TnPP using different detector (a) Gas chromatogram of TnPP by FID; (b) Gas chromatogram of TnPP by FPD

Table S1. Comparison of major component and impurities content at different inlet temperatures

Compounds	260 ℃[1](mg/g)	280 ℃[2](mg/g)	300 ℃[3](mg/g)
Impurity 1	0.05	0.05	0.05
Impurity 2	0.03	0.03	0.03
Impurity 3	0.57	0.62	0.70
Impurity 4	0.28	0.27	0.29
Impurity 5	0.06	0.06	0.07
Impurity 6	0.11	0.12	0.12
TnPP	998.83	998.78	998.66

[1] Inlet temperature is 260 ℃

[2] Inlet temperature is 280 ℃

[3] Inlet temperature is 300 ℃

Table S2. Inorganic impurities content in TnPP

Element	Atomic weight	Content（ng/ml）	Element	Atomic weight	Content（ng/ml）
B	11	24.57	Ni	60	0.12
Na	23	92.98	Cu	63	0.32
Mg	24	605.16	Zn	66	17.54
Al	27	17.73	As	75	0.31
K	39	2.07	Sr	88	0.34
Sc	45	1.97	Sn	118	0.11
Ti	47	97.48	I	127	3.94
V	51	0.26	Ba	137	1.54
Cr	52	2.21	Tb	159	0.01
Mn	55	0.08	W	182	0.02
Fe	56	1.01	U	238	0.01
**Total concentration (ng/ml)**			869.78		
**TnPP (ng/ml)**			1000000		
**Inorganic impurity (mg/g)**			0.870		

Table S3. Purity results of TnPP by MB method

Number	Content (mg/g)	X_W[1] (mg/g)	X_V[2] (mg/g)	X_{NV}[3] (mg/g)	Content (mg/g)	Average value (mg/g)	SD[4] (mg/g)	RSD[5] (%)
1	998.63				994.60			
2	998.63				994.60			
3	998.64				994.61			
4	998.63				994.60			
5	998.62				994.59			
6	998.63	3.16	0.00	0.87	994.60	994.60	0.0081	0.0008
7	998.62				994.59			
8	998.61				994.58			
9	998.63				994.60			
10	998.63				994.60			
11	998.62				994.59			

[1] Water content

[2] Volatile solvent content

[3] Inorganic impurity content

[4] Standard deviation

[5] Relative standard deviation

Table S4. Purity results of TnPP by qNMR

Number	Content of the ^{1}H-qNMR (mg/g)	Content of the ^{31}P-qNMR (mg/g)
1	994.7	992.6
2	992.6	993.7
3	993.6	994.6
4	995.7	994.3
5	994.8	992.0
6	992.7	994.5
7	994.4	992.7
Average value	994.1	993.5
RSD[1]	0.12%	0.11%

[1] Relative standard deviation
